# Polyherbal Medicine Divya Sarva-Kalp-Kwath Ameliorates Persistent Carbon Tetrachloride Induced Biochemical and Pathological Liver Impairments in Wistar Rats and in HepG2 Cells

**DOI:** 10.3389/fphar.2020.00288

**Published:** 2020-03-25

**Authors:** Acharya Balkrishna, Sachin Shridhar Sakat, Ravikant Ranjan, Kheemraj Joshi, Sunil Shukla, Kamal Joshi, Sudeep Verma, Abhishek Gupta, Kunal Bhattacharya, Anurag Varshney

**Affiliations:** ^1^Drug Discovery and Development Division, Patanjali Research Institute, Haridwar, India; ^2^Department of Allied and Applied Sciences, University of Patanjali, Patanjali Yog Peeth, Haridwar, India

**Keywords:** Divya Sarva-Kalp-Kwath, carbon tetrachloride, hepatotoxicity, HepG2 cells, safety, hepatoprotective effects

## Abstract

Divya Sarva-Kalp-Kwath (SKK) is a poly-herbal ayurvedic medicine formulated using plant extracts of *Boerhavia diffusa* L. (Nyctaginaceae), *Phyllanthus niruri* L. (Euphorbiaceae), and *Solanum nigrum* L. (Solanaceae), described to improve liver function and general health. In the present study, we have explored the hepatoprotective effects of SKK in ameliorating carbon tetrachloride (CCl_4_) induced liver toxicity using *in-vitro* and *in-vivo* test systems. Chemical analysis of SKK using Liquid Chromatography-Mass Spectroscopy (LC-MS-QToF) and High-Performance Liquid Chromatography (HPLC) revealed the presence of different bioactive plant metabolites, known to impart hepatoprotective effects. In human hepatocarcinoma (HepG2) cells, co-treatment of SKK with CCl_4_ effectively reduced the hepatotoxicity induced by the latter. These effects were confirmed by studying parameters such as loss of cell viability; release of hepatic injury enzymatic biomarkers- aspartate aminotransferase (AST), and alkaline phosphatase (ALP); and changes in reactive oxygen species and in mitochondrial membrane potentials. *In-vivo* safety analysis in Wistar rats showed no loss in animal body weight, or change in feeding habits after repeated oral dosing of SKK up to 1,000 mg/kg/day for 28 days. Also, no injury-related histopathological changes were observed in the animal's blood, liver, kidney, heart, brain, and lung. Pharmacologically, SKK played a significant role in modulating CCl_4_ induced hepatic injuries in the Wistar rats at a higher dose. In the 9 weeks' study, SKK (200 mg/kg) reduced the CCl_4_ stimulated increase in the release of enzymes (ALT, AST, and ALP), bilirubin, total cholesterol, and uric acid levels in the Wistar rats. It also reduced the CCl_4_ stimulated inflammatory lesions such as liver fibrosis, lymphocytic infiltration, and hyper-plasticity. In conclusion, SKK showed pharmacological effects in improving the CCl_4_ stimulated liver injuries in HepG2 cells and in Wistar rats. Furthermore, no adverse effects were observed up to 10× higher human equivalent dose of SKK during 28-days repeated dose exposure in Wistar rats. Based on the literature search on the identified plant metabolites, SKK was found to act in multiple ways to ameliorate CCl_4_ induced hepatotoxicity. Therefore, polyherbal SKK medicine has shown remarkable potentials as a possible alternative therapeutics for reducing liver toxicity induced by drugs, and other toxins.

## Introduction

Liver is the principal organ involved in the enzymatic metabolism of drugs, xenobiotics, and toxins. Following hepatic expsure, xenobiotics and harsh chemicals such as carbon tetrachloride (CCl_4_; haloalkene) are metabolized by liver microsomal enzyme CYP2E1 into reactive intermediates such as tetrachloromethane radical (${\text{CCl}}_{3}^{•}$) and trichloromethyl peroxy radical (CCl_3_OO^•^) (Abraham and Wilfred, 2002; Nada et al., 2010). These reactive intermediates interact and induce oxidation of the membrane lipids and proteins of hepatic stellate, kupffer, and endothelial cells. These damages lead to the elevation of injury associated biomarkers- aspartate aminotransferase (AST), acid phosphatase (ASP), alkaline phosphatase (ALP), alanine transaminase (ALT), gamma-glutamyl-transferase, lactate dehydrogenase (LDH), glucose, globulin, bilirubin, and cholesterol levels (Yadav and Kumar, 2014). Chronic and long-term injuries can lead to the development of liver cirrhosis (Debnath et al., 2013).

Recent publications have drawn focus on the importance of herbal formulation in modulating complex life-changing diseases such as rheumatoid arthritis and psoriasis (Balkrishna et al., 2019b,c,d). The traditional medicinal system is based on the application of polyherbal formulations. Pharmacological effects obtained from multiple plant extract metabolites are much higher as compared to single herb extracts (Yadav et al., 2008; Balkrishna et al., 2019a). In modern medicine, the treatment for chronic diseases has shifted from the “one drug, one target, one disease” paradigm toward combination therapies (Williamson, 2001; Zhou et al., 2016). Synergistic treatments also significantly reduce possible incidences of health-related side-effects.

Divya Sarva-kalp-kwath (SKK) decoction having a poly-herbal origin has been given for healing acute hepatic diseases and have been found effective in ameliorating CCl_4_ stimulated sub-acute hepatotoxicity in Wistar rats following a 7 days' treatment (Yadav and Kumar, 2014). It is prepared using aqueous extracts from *Boerhavia diffusa* L. (Nyctaginaceae), *Phyllanthus niruri* L. (Euphorbiaceae), and *Solanum nigrum* L. (Solanaceae) plants mixed in the ratio of 2:1:1. These plants have been cited in the traditional medicinal texts to provide protection against hepatic diseases and other injuries (Kirtikar and Basu, 1956). Herbal component of SKK, *B. diffusa* L. plant also known as “Punarnava” possesses a variety of isoflavinoids such as rotenoids, flavonoids, flavonoid glycosides, xanthones, lignans, ecdysteroids, and steroids. Several of these plant metabolites such as rotenoids (Boeravinone A- G), kaempferol, and quercetin have been proven for their role in hepatoprotective activity (Lami et al., 1990; Borrelli et al., 2005; Ferreres et al., 2005; Pereira et al., 2009; Bairwa et al., 2013). *B. diffusa* L. plant extract has been found to modulate CCl_4_ induced liver injury in stimulated animals through the reduction of cytochrome (CYP) enzyme activities (Ramachandra et al., 2011; Venkatesh et al., 2012; Bairwa et al., 2013; Patel and Verma, 2014; Ekow Thomford et al., 2018; Juneja et al., 2020). *P. niruri* L. also known as ‘bhumi amalaki’ is composed of alkaloids, anthrocyanins, chlorogenic acids, flavonoids, lignans, phenolic acids, tannins, terpenoids, saponins, and substitutes that attribute to its bioactivity (Kaur et al., 2017; Jantan et al., 2019). Metabolites present in the plant extract act as antioxidants reducing CCl_4_ stimulated liver injuries in hepatocyte (Syamasundar et al., 1985; Harish and Shivanandappa, 2006; Bhattacharjee and Sil, 2007). *S. nigrum* L. also known as “Makoy” contains several steroidal glycosides, steroidal alkaloids and steroidal oligoglycosides that also act as antioxidants reducing CCl_4_ induced hepatic injuries through amelioration of oxidative stress (Lin et al., 2008; Mir et al., 2010; Elhag et al., 2011; Sivgami et al., 2012).

Therefore, in the present study, we explore the safety and long-term pharmacological effects of SKK decoction with a possible synergistic effect from the presence of three potent plant species having hepatoprotective effects in ameliorating chronic liver injuries induced in Wistar rats following 9 weeks' stimulation with CCl_4_. For the study, we screened, identified and quantified essential metabolites present in the SKK decoction using Liquid Chromatography-Mass Spectroscopy QToF (LC-MS QToF) and High-Performance Liquid Chromatography (HPLC) analytical techniques. Using human hepatocarcinoma (HepG2) cells, we verified the biological effect and mode of action of SKK in ameliorating the chronic CCl_4_ induced cytotoxicity and injuries. We also studied the safety profile of SKK decoction in Wistar rats through the measurement of cellular and biochemical parameters after 28 days of repeated dosing. The pharmacological effect of SKK decoction in reducing CCl_4_ induced chronic toxicity in Wistar rats was explored over 9 weeks' time-period through analysis of the physiological, biochemical, and histopathological changes. As a positive control drug, Silymarin (SLM) treatment was also given to the CCl_4_ stimulated Wistar rats to ameliorate the CCl_4_ induced liver injuries.

## Materials and Methods

### Chemicals and Reagents

SKK was obtained from Divya Pharmacy, Haridwar, India, under its brand name “Divya Sarva-Kalp-Kwath” (Batch no- #A-SKK056). Plant identification of the SKK herbal components was performed at the Council of Scientific and Industrial Research—National Institute of Science Communication and Information Resources (CSIR—NISCAIR), Delhi, India and provided the following plant identification voucher numbers–*Phyllanthus amarus* Schum. & Thonn. Syn. *Phyllanthus fraternus* Webster (NISCAIR/RHMD/Consult/2019/3453-54-30), *P. niruri sensu* Hook. f. (NISCAIR/RHMD/Consult/2019/3453-54-149) and *B. diffusa* L. and *S. nigrum* L. (NISCAIR/RHMD/Consult/2019/3453-54-119). CCl_4_ was purchased from MP Biomedicals India Private Limited Mumbai, India. SLM was purchased from Sigma Aldrich, St. Louis, USA. All the solvents and reagents used for LC-MS QToF and HPLC assays were of high spectroscopy grade and purchased from Merck India Pvt. Ltd., Mumbai, India. Haematoxylin, Potassium Aluminum Sulfate Dodecahydrate, Mercury (II) Oxide red were purchased from Merck India Pvt. Ltd., Mumbai, India. Eosin Yellow and Ferric chloride were purchased from Hi-Media Laboratories, Mumbai, India. Biochemistry reagents were procured from Randox Laboratories Ltd., United Kingdom. Food grade olive oil was purchased from the local market. All other chemicals and reagents used for the tissue processing work were of the highest analytical grade.

### Extract Preparation and Dose Calculation

Animal equivalent doses of SKK for rat studies were estimated based on the body surface area of the animals. The human therapeutic recommended dose of the SKK is 5–10 g of powder boiled in 400 mL of water until approximately 100 mL decoction remains.

Accordingly, 7.5 g (average quantity) of SKK was weighed, and boiled in 400 mL of water until a volume of 100 mL remained. The resultant decoction was dried using lyophilizer, and ~853 mg of powder was obtained. Based on the decoction preparation protocol, the human dose was calculated as 14.21 mg/kg. Human equivalent doses (mg/kg) for rat were calculated by multiplying human dose (mg/kg) by factor 6.2 (Nair and Jacob, 2016) and was estimated at 88.14 mg/kg. Taking a round-off, we considered 100 mg/kg as the human equivalent dose and 200 mg/kg as effective and higher doses, respectively.

### Metabolite Analysis of SKK Decoction

Identification of the metabolites present in the SKK decoction was performed using a Xevo G2-XS QToF with Acquity UPLC- I Class and Unifi software (Waters, MA, USA). Separation of the metabolites was performed using an Acquity UPLC HSS -T3 column (100 x 2.1 mm i.d., 1.7 μm). The elution was carried out at a flow rate of 0.4 mL/min using gradient elution of mobile phase 0.1% formic acid in water (mobile phase A) and 0.1 % formic acid in acetonitrile (mobile phase B). Two microliters of the final test solution were injected during the analysis and record the chromatograph for 15 min. The gradient program was set between 0 and 18 min retention time with a flow of 0.4 mL/min.

The LC-MS equipment was equipped with an ESI ion source operating in positive and negative ion mode. A mass range of 50–1,000 Da was set with a 0.2 s scan time, the acquisition time of 15 min, the capillary voltage of 1 kV (for positive mode) and 2 kV (for negative mode). Mass was corrected during acquisition using an external reference (Lock-Spray) consisting of a 0.2 ng/mL solution of leucine-enkephalin.

Quantitative analysis of SKK decoction was performed using Waters HPLC equipped with a Binary pump (1525), photodiode detector array (2998), and auto-sampler (2707). Elution was performed at a flow rate of 1 mL/min using a gradient elution of mobile phase A (0.1% Orthophosphoric acid, pH-2.5 with Diethylamine) and mobile phase B (Acetonitrile). For gallic acid, catechin, caffeic acid, rutin, and quercetin, the solvent gradient program selected was 5% of mobile phase B from 0 to 5 min, 5 to 100% of mobile phase B from 5 to 35 min, 100% of mobile phase B from 35 to 45 min, 100 to 50% of mobile phase B from 45 to 47 min, 50 to 5% of mobile phase B from 47 to 48 min, 5% of mobile phase B from 48 to 55 min. For corilagin, the solvent gradient program selected was 5% of mobile phase B from 0 to 5 min, 5 to 20% of mobile phase B from 5 to 20 min, 20 to 30% of mobile phase B from 20 to 25 min, 30 to 75% of mobile phase B from 25 to 30 min, 75 to 90% of mobile phase B from 30 to 35 min, 90% of mobile phase B from 35 to 40 min, 90 to 5% of mobile phase B from 40 to 41 min, 5% of mobile phase B from 41 to 45 min. The X-Bridge Phenyl Column (5 μm, 4.6 × 250 mm) was used for gallic acid, catechin, caffeic acid, rutin and quercetin, and Shodex C18-4E column (5 μm, 4.6 × 250 mm) was used for corilagin. Detector wavelength was kept at 270 nm for gallic acid- catechin- corilagin and at 325 nm for caffeic acid- rutin- quercetin. The column temperature was maintained 35°C and 10 μL of test solution was injected during the analysis.

### *In-vitro* Biological Effect of SKK in Ameliorating CCl_4_ Induced Injury

HepG2 cells were sourced from the ATCC authorized cell repository, National Center for Cell Sciences, Pune, India and grown in Dulbecco's Modified Eagle Hi-glucose medium supplemented with 10% fetal bovine serum and 1% penicillin-streptomycin antibiotics at 37°C and 5% CO_2_ under humid conditions. For the experiments, the cells were seeded in 96 and 24 well plates at a density of 1 × 10^5^ cells/mL. The cells were pre-incubated overnight. The next day, they were pre-treated for 1 h with SKK at final concentrations of 0.25, 0.5, and 1 mg/mL and then with 10 mM CCl_4._ The exposed cells were incubated further for 18 h. At the end of the treatment time period, the exposure medium was replaced with 100 μL of fresh media containing 0.5 mg/mL of 3-(4,5-dimethylthiazol-2-yl)-2,5-diphenyltetrazolium bromide (MTT) in each well. The plates were incubated for an additional 3 h at 37°C and at the end of the time period, 50 of 75 μL of MTT containing media was removed from each well and 50 μL of pure DMSO was added. The plates were placed on a shaker for 10 min at 200 rpm. The absorbance of each well was recorded using the PerkinElmer Envision microplate reader, at 595 nm wavelength and cell viability percentage was calculated. For the analysis of AST, and ALT released from the HepG2 cells, the supernatant was collected from 24 well plates exposed to SKK and CCl_4_ following the method mentioned for MTT and analyzed using Randox chemical analyzer (RX Monaco) and associated standard test kits (Randox Laboratories Ltd., United Kingdom).

For the detection of reactive oxygen species (ROS) and mitochondrial membrane potential (MMP), the HepG2 cells were seeded on the transparent bottom 96 well black plate at a density of 5 × 10^5^ cells/mL and pre-incubated as mentioned above. The next day, cells were pre-treated with SKK at a final concentration of 1 mg/mL. After 1 h, cells were induced with CCl_4_ (10 mM) for 12 h treatment. For measuring ROS and MMP, CellROX, and Mitotracker Red reagents (Thermo Fisher) were added, respectively, following the manufacturer's protocols. High-content analysis (HCA) of the cells was performed using a Nikon epifluorescence microscope, and images were analyzed using the HCS Studio software suite.

### Experimental Animal Model

Male albino rats of Wistar strain (body weight 180–200 g) were used for the safety and pharmacological studies. Animals were procured from Liveon Life Sciences Pvt. Ltd., India and were housed in polypropylene cages in controlled room temperature 22 ± 2°C and relative humidity of 60–70% with 12:12 h light and dark cycle in a registered animal house (1964/PO/Rc/S/17/CPCSEA). All animals were fed standard nutritionally balanced pellet diet (Purina Lab Diet, St. Louis, MO, USA) and sterile filtered water *ad libitum*. Animal experimentation ethical clearance was obtained from the Institutional Animal Ethical Committee of the Patanjali Research Institute, Haridwar, India. For the study, standard operating procedures and protocols were followed according to the approval numbers: PRIAS/LAF/IAEC-048 and PRIAS/LAF/IAEC-007.

### Grouping of Experimental Animals

For the safety assessment, animals were randomized and divided into four treatment groups (*n* = 5 animals per group).

Group 1: Normal control (NC) animals were administered with distilled water only.
Group 2: SKK doses of 100 mg/kg.
Group 3: SKK dose of 500 mg/kg.
Group 4: SKK dose of 1,000 mg/kg.

For the pharmacological study, animals were divided randomly into five groups (*n* = 7 animals per group):

Group 1: NC animals administered with olive oil (intraperitoneal injection; 0.25 ml/kg; every 3rd day for 9 weeks) and 0.25% Na-CMC orally.
Group 2: Disease control (DC) animals administered with CCl_4_ in olive oil v/v (intraperitoneal injection; 0.5 ml/kg; every 3rd day for 9 weeks).
Group 3: Animals were administered with CCl_4_ in olive oil v/v (intraperitoneal injection; 0.5 ml/kg; every 3rd day for 9 weeks), and concurrent oral treatment of SLM (100 mg/kg; once daily for 9 weeks).
Group 4: Animals were administered with CCl_4_ in olive oil v/v (intraperitoneal injection; 0.5 ml/kg; every 3rd day for 9 weeks), and concurrent oral treatment of SKK (100 mg/kg; once daily for 9 weeks).
Group 5: Animals were administered with CCl_4_ in olive oil v/v (intraperitoneal injection; 0.5 ml/kg; every 3rd day for 9 weeks), and concurrent oral treatment of SKK (200 mg/kg; once daily for 9 weeks).

For the safety study, all SKK doses were dissolved in distilled water and given once a day through gavage administration for 28 days. In the case of pharmacological study, fresh suspension of SLM (using 0.25% Na-CMC) and solution of SKK were prepared daily and administered to the corresponding group of animals at the dose volume of 10 ml/kg. The animals were observed for body weight changes daily throughout the study period and averaged on a per-day basis for the safety study, whereas for the pharmacological study, they were averaged per week. Similarly, animal feed and water consumption were recorded daily until the end of the experiment. Histopathological, pathological, and biochemical changes in the animals were analyzed at the end of the study period following the euthanization of the animals.

#### Hematological and Serum Biochemical Analysis

Whole blood samples were collected from all the rats using the tail-snip method. In the safety study, parameters such as hemoglobin (Hb), total red blood corpuscles (RBC), hemoglobin per RBC (MCH), mean corpuscular hemoglobin concentration per unit volume (MCHC), mean corpuscular size (MCV), the total and differential counts of leukocytes were analyzed in the whole blood samples using the hematology analyzer BC-2800 (Mindray, Haryana, India).

In both the safety and pharmacological effect studies, analysis of biochemical parameters in the blood serum such as aspartate aminotransferase (AST), and alanine aminotransferase (ALT), urea, albumin, total bilirubin, total cholesterol, and creatinine levels were performed following manufacturer's protocol using Randox chemical analyser (RX Monaco) and associated standard test kits (Randox Laboratories Ltd, United Kingdom).

#### Histopathological Evaluation

The euthanized animals were dissected, and their target organs were excised. A portion of each tissue was fixed at 10% buffered formalin and embedded in paraffin. Solid sections of 5 μm thickness were made using a microtome and stained with hematoxylin-eosin (H&E). Blinded histopathological analysis of the H&E stained tissue sections was done by a veterinarian pathologist using an Olympus Magnus microscope camera. For the safety study, liver and kidney tissue samples were analyzed for determining the presence and distribution of focal, multifocal, and diffuse lesions. For the pharmacological effect study, only the liver tissue samples were analyzed for severity of the lesions that were scored as 0 = Not Present, 1 = Minimal (<1%), 2 = Mild (1–25%), 3 = Moderate (26–50%), 4 = Moderately Severe (51–75%), 5 = Severe (76–100%). Average of the individual scores obtained from all the animals present in a group were calculated to generate each lesion score. Finally, the mean of respective tissue lesions score were considered for the final calculations.

### Statistical Analysis

Data are expressed as mean ± standard error of means (SEM) for each group. A one-way analysis of variance (ANOVA) followed by Dunnett's multiple comparison *t*-test was used to calculate the statistical difference. Values of *p* < 0.05 were considered statistically significant. Statistical analysis was done using GraphPad Prism version 7.0 software (GraphPad Software, CA).

## Results

### LC-MS-QToF and HPLC Based Metabolite Analysis of SKK

**Figure 1:**
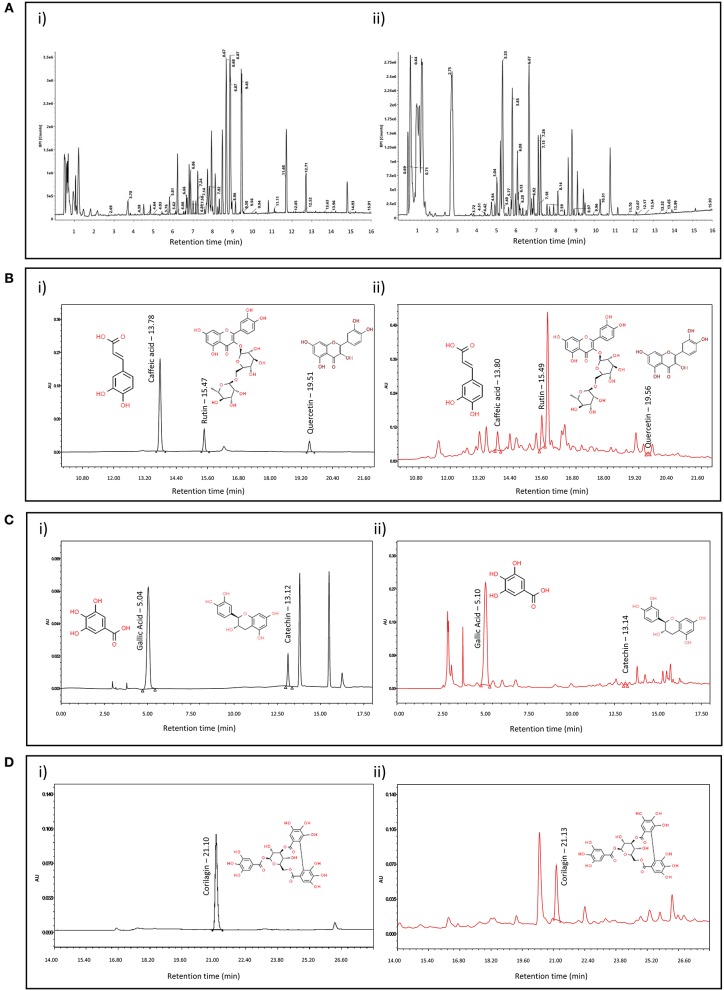
Plant metabolite analysis of Divya Sarva-Kalp-Kwath (SKK): Aqueous extract of the SKK was screened for plant metabolites using liquid chromatography (LC)-based mass spectroscopy (MS). Spectral analysis revealed the presence of (**A**, i) 46 metabolites were detected in the positive mode and (**A**, ii) 48 metabolites were detected in the negative mode at different retention time (RT; min) (see Table 1). Using High Performance Liquid Chromatography (HPLC) analysis, SKK was analyzed and quantified for the metabolites, (**B**, i) Standards of caffeic acid, rutin, and quercetin, (**B**, ii) SKK contents of caffeic acid, rutin, and quercetin detected at 325 nm, (**C**, i) standards of gallic acid and catechin, (**C**, ii) SKK contents of gallic acid and catechin detected at 270 nm, and (**D**, i) standards of corilagin, (**D**, ii) SKK contents of corilagin detected at 270 nm. Gallic acid (RT: 5.10 min), catechin (RT: 13.14 min), caffeic acid (RT: 13.80 min), rutin (RT: 15.50 min), quercetin (RT: 19.56 min), and corilagin (RT: 21.13 min) were detected at their respective retention time (RT) in the developed and validated analytical HPLC method.

Aqueous extract of the SKK was first analyzed using LC-MS QToF technique. Results showed the presence of 68 identifiable metabolites present in SKK decoction. Among these, 46 metabolites were detected in the positive mode and 48 metabolites in the negative mode (Figure 1Ai,ii). Metabolites identified were citric acid monohydrate, gallic acid, gentisic acid, catechin, brevifolincarboxylic acid, caffeic acid, corilagin, rutin, ellagic acid, naringenin, apigenin, quercetin, kaempferol, solasodine, and coccineone B as a major metabolites. Based on a literature search, sources of these metabolites were identified as, *B. diffusa* L., *P. niruri* L., and *S. nigrum* L. (Table 1). Furthermore, the spectral signature of several other metabolites was also identified in the SKK decoction which is currently under investigation.

**Table 1:** Liquid chromatography based metabolite analysis of the aqueous extract of Divya Sarva-Kalp-Kwath (SKK) showed the presence of 68 metabolites.

**S.No**.	**Component name**	**Formula**	**RT (min)**	**Ionization mode**	**Observed m/z**	**Plant species**
1	Citric acid monohydrate	C_6_H_10_O_8_	0.64	–ve	209.0304	*Solanum nigrum* L. (Atanu et al., 2011; Albouchi et al., 2018)
2	Tartaric acid	C_4_H_6_O_6_	0.69	–ve	149.0095	*Solanum nigrum* L. (Atanu et al., 2011)
3	D-(-)-Quinic acid	C_7_H_12_O_6_	0.71	–ve	191.0567	
4	Gallic acid	C_7_H_6_O_5_	2.69/2.75	+ve/–ve	171.0295/169.0141	*Solanum nigrum* L., *Phyllanthus* species (Huang et al., 2010; Mao et al., 2016; Jantan et al., 2019)
5	Gentiatibetine	C_9_H_11_NO_2_	3.7/3.72	+ve/–ve	166.0873/164.0717	
6	Pseudolaroside C	C_14_H_18_O_8_	4.38	+ve	315.1059	
7	Gentisic acid	C_7_H_6_O_4_	4.42	–ve	153.0195	*Phyllanthus* species (Huang et al., 2010)
8	Gentianine	C_10_H_9_NO_2_	4.43	+ve	176.0715	
9	Koaburaside	C_14_H_20_O_9_	4.51	–ve	331.1027	*Phyllanthus* species (Mao et al., 2016)
10	Pyrogallic acid	C_6_H_6_O_3_	4.64	+ve	127.0399	*Phyllanthus* species (Mao et al., 2016)
11	3-O-trans-Coumaroylquinic acid	C_16_H_18_O_8_	4.66	+ve	339.1057	*Solanum* species (Daji et al., 2018)
12	Cornoside	C_14_H_20_O_8_	4.67	–ve	315.1076	
13	1-O-Caffeoyquinic acid	C_16_H_18_O_9_	4.93/4.94	+ve/–ve	355.1034	*Phyllanthus* species (Mao et al., 2016)
14	Picrasidine V	C_12_H_8_N_2_O_4_	5.03/5.04	+ve/–ve	245.0562/243.0439	
15	Demethylcoclaurine	C_16_H_17_NO_3_	5.13	+ve	272.1285	
16	Camphoronic Acid	C_9_H_14_O_6_	5.32/5.33	+ve/–ve	219.087/217.0712	
17	Protocatechuic aldehyde	C_7_H_6_O_3_	5.4	–ve	137.0245	*Solanum* species (Kaunda and Zhang, 2019)
18	5-Caffeoylquinic acid	C_16_H_18_O_9_	5.62/5.64	+ve/–ve	355.103/353.0871	*Solanum* species (Daji et al., 2018)
19	Umbelliferone	C_9_H_6_O_3_	5.62	+ve	163.04	
20	Catechin	C_15_H_14_O_6_	5.67	–ve	289.0712	*Phyllanthus* species (Huang et al., 2010; Mao et al., 2016)
21	Chlorogenic acid	C_16_H_18_O_9_	5.76/5.77	+ve/–ve	355.104/353.087	*Solanum nigrum* L., *Phyllanthus* species (Huang et al., 2010; Mao et al., 2016)
22	Brevifolincarboxylic acid	C_13_H_8_O_8_	5.81/5.83	+ve/–ve	293.0298/291.0142	*Phyllanthus* species (Mao et al., 2016)
23	Caffeic acid	C_9_H_8_O_4_	6.02	–ve	179.0347	*Solanum nigrum* L. (Huang et al., 2010)
24	Phyllanthusiin E	C_13_H_8_O_8_	6.06/6.08	+ve/–ve	293.0296/291.014	
25	Corilagin	C_27_H_22_O_18_	6.13	–ve	633.074	*Phyllanthus* species (Mao et al., 2016)
26	Gallocatechin	C_15_H_14_O_7_	6.2	–ve	305.0695	*Phyllanthus* species (Mao et al., 2016)
27	Coumurrin	C_16_H_18_O_6_	6.27	–ve	305.1049	
28	Brevifolin	C_12_H_8_O_6_	6.66/6.67	+ve/–ve	249.0401/247.0243	*Phyllanthus* species (Mao et al., 2016)
29	Quercetin 3,7-diglucoside	C_27_H_30_O_17_	6.73/6.74	+ve/–ve	627.1566/625.1418	
30	Evodionol	C_14_H_16_O_4_	6.86	+ve	249.1125	
31	Kaempferol-3-O-rutinoside	C_27_H_30_O_15_	6.89	+ve	595.164	*Solanum* species, *Boerhavia diffusa* L. (Daji et al., 2018; Kumar et al., 2018)
32	m-coumaric acid	C_9_H_8_O_3_	6.92	–ve	163.0401	*Solanum nigrum* L. (Huang et al., 2010)
33	Rutin	C_27_H_30_O_16_	7.14/7.15	+ve/–ve	611.1609/609.1468	*Solanum nigrum* L., *Phyllanthus* species, *Solanum* species (Huang et al., 2010; Mao et al., 2016; Daji et al., 2018)
34	Citrusin B	C_27_H_36_O_13_	7.18	–ve	567.2089	*Solanum* species (De Souza et al., 2019)
35	Ellagic Acid	C_14_H_6_O_8_	7.24/7.26	+ve/–ve	303.0142/300.9985	*Phyllanthus* species (Mao et al., 2016)
36	Myricitrin	C_21_H_20_O_12_	7.34/7.35	+ve/–ve	465.1024/463.0879	*Solanum nigrum* L. (Huang et al., 2010)
37	(3R)-Abruquinone B	C_20_H_22_O_8_	7.37	–ve	389.1235	
38	Ecdysterone	C_27_H_44_O_7_	7.42	+ve	481.3165	
39	Kaempferol-3-o-beta-glucopyranosyl7-o-alpha-rhamnopyranoside	C_27_H_30_O_15_	7.58/7.59	+ve/–ve	595.1668/593.1516	
40	Astragalin	C_21_H_20_O_11_	7.81	–ve	447.0911	*Phyllanthus* species (Mao et al., 2016)
41	Solamargine	C_45_H_73_NO_15_	7.82	+ve	868.5101	*Solanum* species (Daji et al., 2018)
42	Dipropylmalonic acid	C_9_H_16_O_4_	8.14	–ve	187.0973	
43	Naringenin	C_15_H_12_O_5_	8.19/8.21	+ve/–ve	273.0765/271.0601	*Solanum nigrum* L. (Huang et al., 2010)
44	Rhoifolin	C_27_H_30_O_14_	8.36	+ve	579.1694	*Solanum* species (Refaat et al., 2015)
45	Apigenin	C_15_H_10_O_5_	8.92	–ve	269.0454	*Solanum nigrum* L. (Huang et al., 2010)
46	Chuanbeinone	C_27_H_43_NO_2_	8.67	+ve	414.3369	
47	Solasonin	C_45_H_73_NO_16_	8.68	+ve	884.5064	*Solanum* species (Daji et al., 2018)
48	Eupalitin 3-galactoside	C_23_H_24_O_12_	8.79/8.8	+ve/–ve	493.1346/491.12	*Boerhavia diffusa* L. (Pereira et al., 2009)
49	Solasodine glucoside	C_33_H_53_NO_7_	8.87	+ve	576.3908	
50	Solanine	C_45_H_73_NO_15_	8.87	+ve	868.5114	*Solanum nigrum* L. (Albouchi et al., 2018)
51	Eupalitin-3-O-b-D-glucoside	C_23_H_24_O_12_	8.93	–ve	491.1198	*Boerhavia diffusa* L. (Pereira et al., 2009)
52	Moupinamide	C_18_H_19_NO_4_	8.96/8.97	+ve/–ve	314.139/312.1236	
53	Quercetin	C_15_H_10_O_7_	9.3/9.32	+ve/–ve	303.0501/301.0348	*Solanum nigrum* L. (Huang et al., 2010; Mishra et al., 2014)
54	Solasodine 3-b-D-glucopyranoside	C_33_H_53_NO_7_	9.45	+ve	576.3906	*Solanum* species (Li et al., 2015)
55	Apigenin-7-O-α-L-rhamnose(1 → 4)-6"-O-acetyl-β-D-glucoside	C_29_H_32_O_15_	9.56	+ve	621.1809	
56	Butein	C_15_H_12_O_5_	9.94/9.96	+ve/–ve	273.0765/271.0608	*Solanum* species (Bovy et al., 2007)
57	Kaempferol	C_15_H_10_O_6_	10.31	–ve	285.0402	*Solanum nigrum* L., *Boerhavia diffusa* L. (Huang et al., 2010; Mishra et al., 2014)
58	Solasodine	C_27_H_43_NO_2_	11.11	+ve	414.3364	*Solanum nigrum* L. (Albouchi et al., 2018)
59	Eupalitin	C_17_H_14_O_7_	11.68/11.7	+ve/–ve	331.0814/329.0662	*Boerhavia diffusa* L. (Pandey et al., 2005)
60	Coccineone B	C_16_H_10_O_6_	12.05/12.07	+ve/–ve	299.056/297.0399	*Boerhavia diffusa* L. (Mishra et al., 2014)
61	Boeravinone E	C_17_H_12_O_7_	12.17	–ve	327.0508	*Boerhavia diffusa* L. (Lami et al., 1990; Bairwa et al., 2013; Mishra et al., 2014)
62	Boeravinone K	C_17_H_12_O_6_	12.52/12.54	+ve/–ve	313.0714/311.0559	*Boerhavia diffusa* L. (Bairwa et al., 2013)
63	Veratramine	C_27_H_39_NO_2_	12.71	+ve	410.3059	
64	Boeravinone I	C_18_H_14_O_7_	13.32	–ve	341.0664	*Boerhavia diffusa* L. (Mishra et al., 2014)
65	Boeravinone B	C_17_H_12_O_6_	13.63/13.65	+ve/–ve	313.0713/311.0552	*Boerhavia diffusa* L. (Bairwa et al., 2013; Mishra et al., 2014)
66	Boeravinone G	C_18_H_14_O_7_	13.96/13.99	+ve/–ve	343.0856/341.066	*Boerhavia diffusa* L. (Mishra et al., 2014)
67	Tigogenin	C_27_H_44_O_3_	14.93	+ve	417.3361	*Solanum nigrum* L. (Albouchi et al., 2018)
68	Boeravinone A	C_18_H_14_O_6_	15.91/15.93	+ve/–ve	327.0872/325.071	*Boerhavia diffusa* L. (Mishra et al., 2014)

*Out of these 46 metabolites were detected in the positive mode. Forty-eight metabolites were detected in the negative mode (see Figure 1). Based on a literature search, sources of these metabolites were identified as- (a) Boerhavia diffusa L., (b) Phyllanthus niruri L., and (c) Solanum nigrum L. and listed on the basis of their retention time (RT; min), respective charge and observed mass to charge (m/z) ratio*.

Quantitative analysis of selected metabolite present in the SKK decoction was performed using HPLC method and confirmed the presence of gallic acid (RT: 5.10 min), catechin (RT: 13.14 min), caffeic acid (RT: 13.80 min), rutin (RT: 15.49 min), quercetin (RT: 19.56 min), corilagin (RT: 21.13 min; Figures 1B–D). Using internal standards, the metabolites were quantified as, gallic acid: 1.929 μg/mg, catechin: 0.197 μg/mg, rutin: 0.223 μg/mg, caffeic acid: 0.028 μg/mg, quercetin 0.011 μg/mg, and corilagin 2.954 μg/mg (Figures 1Bi,ii,Ci,ii,Di,ii).

### *In-vitro* Biological Effect Study of SKK Decoction in Reducing CCl_4_ Stimulated Hepatotoxicity

**Figure 2:**
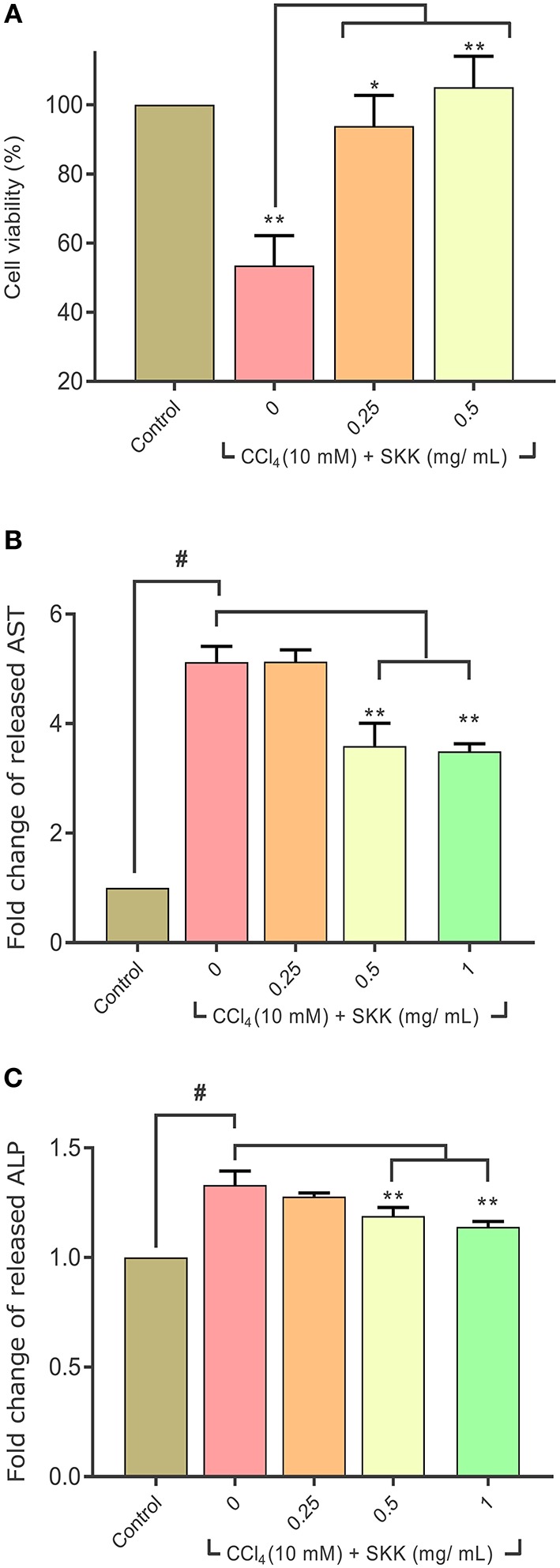
*In-vitro* analysis of cellular and biochemical effects of Divya Sarva-Kalp-Kwath (SKK): **(A)** significant loss of cell viability was observed following the stimulation of HepG2 cells with 10 mM of CCl_4_. Co-treatment of the CCl_4_ stimulated HepG2 cells with SKK led to a significant recovery of cell viability. Also, CCl_4_ stimulation of the HepG2 cells led to an augmented release of liver injury serum-based biomarkers, **(B)** aspartate aminotransferase (AST), and **(C)** alkaline phosphatase (ALP). Release of these serum hepatotoxicity biomarkers was ameliorated following co-treatment of the CCl_4_ stimulated HepG2 cells with SKK (0.5 and 1 mg/mL). Results are expressed as mean ± standard deviation. One-way analysis of variance (ANOVA) followed by Dunnett's multiple comparison *t*-test was used to calculate the statistical difference. *p*-value *<0.05; **<0.01; ^#^<0.01.

Under *in-vitro* conditions, stimulation of the HepG2 cells with 10 mM carbon tetrachloride (CCl_4_), induced a significant (*p* < 0.01) loss of cell viability (53 ± 9%) (Figure 2A). Treatment of these CCl_4_ stimulated HepG2 cells with SKK at the concentrations of 0.25 and 0.5 mg/mL significantly reversed the loss in cell viability to 94 ± 9% (*p* < 0.05) and 105 ± 9% (*p* < 0.01), respectively. Analysis of the released AST and ALP enzymatic biomarkers in the CCl_4_ stimulated HepG2 cell culture supernatant showed a ~1.5–2-folds' increase, compared to the untreated normal control (NC) cells (Figures 2B,C). Treatment of the CCl_4_ stimulated HepG2 cells with SKK (0.5 and 1 mg/mL) significantly (*p* < 0.01) attenuated the release of the liver injury AST and ALP biomarkers (Figures 2B,C). SLM treatment of the CCl_4_ stimulated HepG2 cells also showed a reversal of the CCl_4_ induced cytotoxicity and release of hepatotoxicity biomarkers (data not shown).

**Figure 3:**
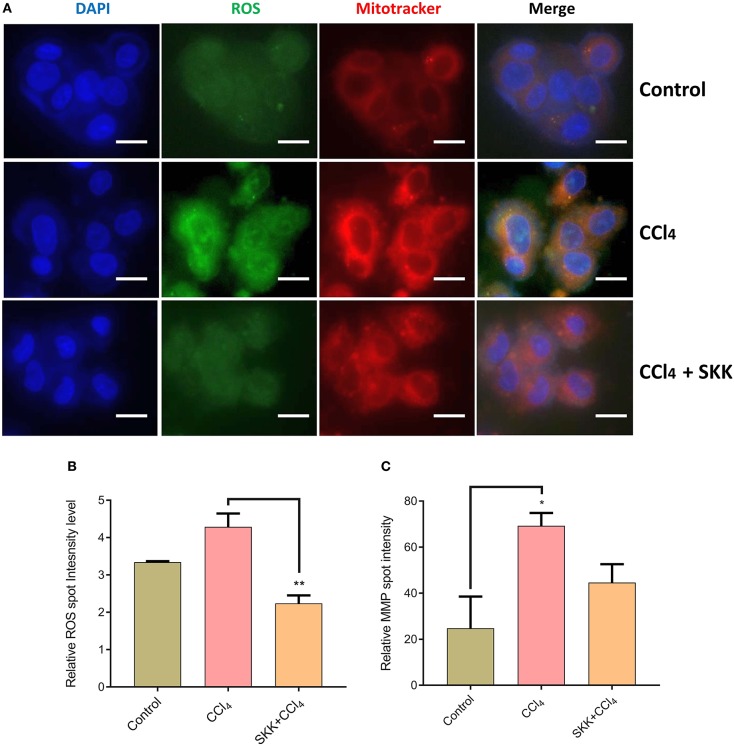
Reactive oxygen species and mitochondrial membrane potential measurement in Divya Sarva-Kalp-Kwath (SKK) and carbon tetrachloride (CCl_4_) treated HepG2 cells: **(A)** Oxidative stress was induced in the HepG2 cells following stimulation with the CCl_4_. Intracellular presence of reactive oxygen species (ROS; green color) and an increase in mitochondrial membrane potential (MMP; red color) were determined through epifluorescence microscope based imaging and HCS Studio software based analysis. Co-treatment of the HepG2 cells with SKK and CCl_4_ significantly reduced the production of ROS and MMP. **(B)** Quantitatively, ROS levels in the CCl_4_ stimulated HepG2 cells showed an upregulation. **(C)** Similarly, MMP in the CCl_4_ stimulated HepG2 cells showed an upregulation. Both these parameters showed a reduction following the co-treatment of the HepG2 cells with CCl_4_ and SKK. Results are expressed as Mean ± Standard Error of Means. One-way analysis of variance (ANOVA) followed by Dunnett's multiple comparison *t*-test was used to calculate the statistical difference. *p*-value *<0.05; **<0.01. Bars represent 200 μm.

High content analysis (HCA) of the CCl_4_ stimulated HepG2 cells was done to study the modulation of ROS and MMP following treatment with SKK (1 mg/mL) (Figure 3A). The representative figures showed an expression of ROS in green colored fluorescence and MMP in red-colored fluorescence; the merged fluorescence images produced yellow-colored spots due to co-localization within the HepG2 cells (Figure 3A). Quantitative analysis of the microscopic images showed a high induction of ROS levels in the HepG2 cells following stimulation with CCl_4_ (10 mM) (Figure 3B). Treatment of these stimulated cells with SKK (1 mg/mL) led to a significant (*p* < 0.01) reduction in the intracellular release of ROS. Similarly, enhanced MMP levels in the CCl_4_ stimulated HepG2 cells as compared to control cells, were also found to be significantly reduced (*p* < 0.05) following treatment with SKK decoction (Figure 3C).

### *In-vivo* Safety of SKK Decoction

**Figure 4:**
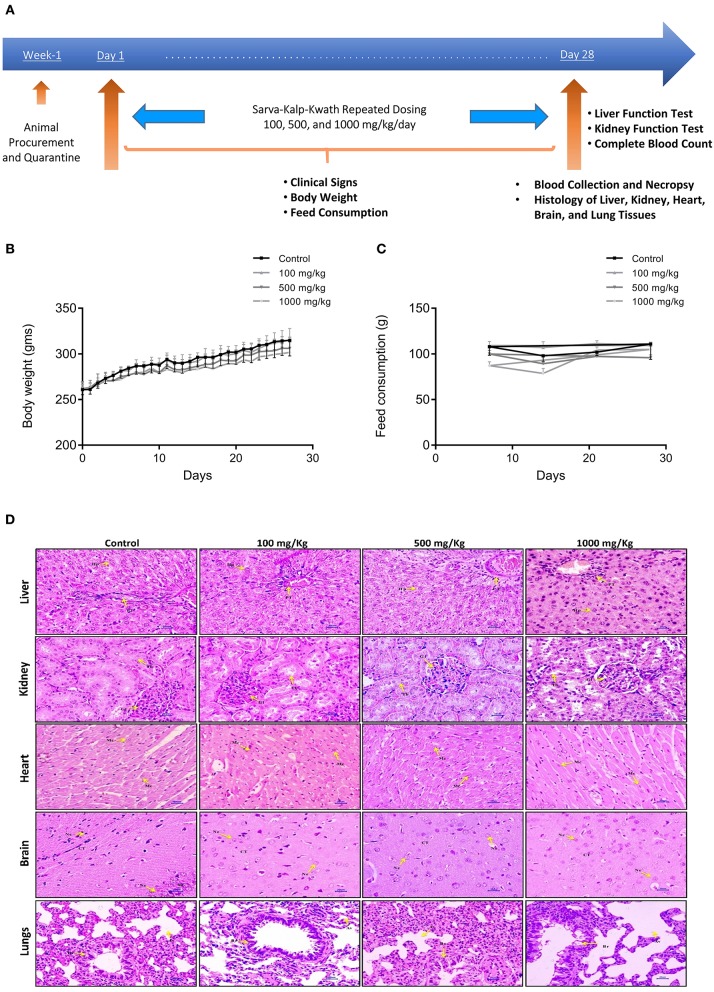
*In-vivo* Safety Analysis of Divya Sarva-Kalp-Kwath (SKK): **(A)** Male Wistar rats were randomized and divided into four treatment groups (*n* = 5): Group 1: Normal control (NC) animals were administered with distilled water only; Group 2: SKK doses of 100 mg/kg; Group 3: SKK dose of 500 mg/kg; and Group 4: SKK dose of 1,000 mg/kg. Water extract of SKK was dissolved in distilled water and administered daily by gavage for 28 days. The animals were observed for physiological, histopathological, and biochemical changes. **(B)** Body weight change in the SKK exposed animals was measured daily and no changes were detected throughout the study period. **(C)** Feed consumption of the SKK treated animals was determined daily and averaged per week. The animals did not show any change in their feed consumption. Histopathological analysis of the **(D)** Liver, kidney, heart, brain, and lung tissue samples obtained from the Wistar rats, did not show any pathological lesions following exposure- Control, SKK 100 mg/kg, SKK 500 mg/ml, and SKK 1,000 mg/kg treatment. Results are expressed as a Mean ± Standard Error of Means. One-way analysis of variance (ANOVA) followed by Dunnett's multiple comparison *t*-test was used to calculate the statistical difference. No statistical significance was detected.

In a 28 days repeat dose safety study, no change was observed in the daily body weight of the Wistar rats treated with SKK decoction at the concentrations of 100, 500, and 1,000 mg/kg/day (Figures 4A,B). Similarly, no variation was observed in the daily average feed consumption of the different animal groups throughout the study period (Figure 4C). Histopathological analysis of the lung, brain, heart, liver and kidney tissues in the SKK treated rats showed the absence of any focal, multifocal, or diffuse inflammatory lesions (Figure 4D). Whole blood analysis also showed no change in the levels of hemoglobin, red blood cells (RBC) indices, total and differential leucocyte counts in the SKK-treated animals (Table 2). Similarly, albumin, glucose, AST, ALT (*p* < 0.05 at 1,000 mg kg day), ALP, total bilirubin urea, creatinine, and total cholesterol levels remained unchanged in the serum of the SKK treated groups, compared with the NC animals (Tables 2, 3). Thus, 28 days treatment of the Wistar rats with SKK did not produce any adverse effects up to the dose of 1,000 mg/kg/day.

**Table 2:** Hematological safety analysis of 28 days repeated dosing of SKK in male Wistar rats: Parameters such as hemoglobin (Hb), total red blood corpuscles (RBC), hemoglobin per RBC (MCH), mean corpuscular hemoglobin concentration per unit volume (MCHC), mean corpuscular size (MCV), the total and differential counts of leukocytes were tested in the whole blood collected from the Wistar rats exposed to Divya Sarva-Kalp-Kwath (SKK) decoction up to the concentration of 1,000 mg/kg.

**Gr. no**.	**Dose (mg/kg)**	**Hb (g/dL)**	**Total RBC (×10^**6**^/mm^**3**^)**	**RBC indices**			**Total WBC (×10^**3**^/mm^**3**^)**	**Differential leucocyte count (%)**				
				**MCH (pg/cell)**	**MCV (fL/cell)**	**MCHC (g/dL)**		**Neutrophil**	**Lymphocyte**	**Eosinophil**	**Macrophage**	**Basophil**
1	0	16 ± 1	7.8	21 ± 1	53 ± 3	39	16 ± 5	21 ± 8	73 ± 9	1	4 ± 2	1
2	100	16	7.7	21 ± 1	53 ± 2	39 ± 1	14 ± 6	22 ± 6	70 ± 6	2	4 ± 1	1
3	500	16 ± 1	7.5	10 ± 2	54 ± 1	39 ± 1	15 ± 5	24 ± 5	70 ± 5	1	3 ± 1	2
4	1,000	15 ± 4	7 ± 1	21 ± 1	53 ± 1	39 ± 1	15 ± 4	20 ± 5	72 ± 5	2 ± 2	5 ± 2	1

*No changes were observed in the SKK treated animals in comparison to the normal control animals (0 mg/kg). The results indicated that SKK was safe up to five times the human equivalent oral dose given to the CCl_4_ treated Wistar rats in the pharmacological effect study*.

**Table 3:** Effect of 28-days oral exposure of SKK on blood biochemistry in wistar rats: Biochemical parameters such as urea, creatine, albumin, alanine transaminase (ALT), aspartate aminotransferase (AST), alkaline phosphatase (ALP), total bilirubin, glucose, and cholesterol levels were determined in the serum of Wistar rats exposed to varying concentrations of the Divya Sarva-Kalp-Kwath (SKK).

**Gr. no**.	**Dose (mg/kg)**	**Urea (mg/dL)**	**Creatinine (mg/dL)**	**Albumin (g/dL)**	**ALT (U/L)**	**AST (U/L)**	**ALP (U/L)**	**Total Bilirubin (mg/dL)**	**Glucose (mg/dL)**	**Total Cholesterol (mg/dL)**
1	0	53 ± 3	1	4	112 ± 15	288 ± 36	446 ± 100	0.04 ± 0.05	103 ± 5	63 ± 4
2	100	48 ± 4	0.6	3	109 ± 22	264 ± 24	464 ± 100	0.04 ± 0.09	101 ± 7	54 ± 6
3	500	51 ± 4	0.5	3	114 ± 18	224 ± 36	387 ± 79	0.04 ± 0.05	102 ± 8	56 ± 11
4	1,000	48 ± 7	0.5	3	115 ± 1	199 ± 47	439 ± 137	0.06 ± 0.09	100 ± 6	57 ± 10

*Based on the analysis, no change in these serum-based biochemical parameters was observed in the animals exposed up to the dose of 1,000 mg/kg of SKK in comparison to the normal control animals (0 mg/kg). No changes were observed in the SKK treated animals in comparison to the normal control animals (0 mg/kg). The results indicated that SKK was safe up to five times the human equivalent oral dose given to the CCl_4_ treated Wistar rats in the pharmacological effect study*.

### *In-vivo* Pharmacological Effects of SKK Decoction in Modulating CCl_4_ Induced Hepatotoxicity

**Figure 5:**
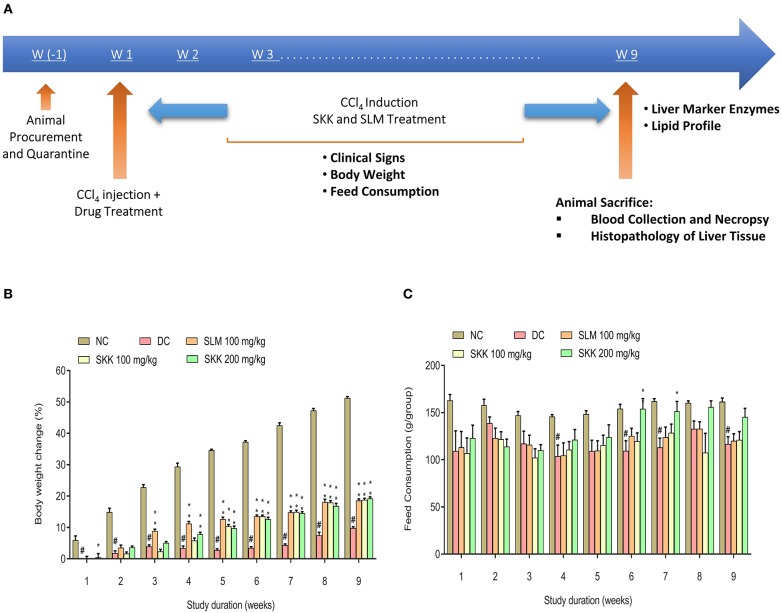
*In-vivo* pharmacological effect study of Divya Sarva-Kalp-Kwath (SKK) in carbon tetrachloride (CCl_4_) treated wistar Rats: **(A)** Wistar rats were divided randomly into four groups (*n* = 5): Group 1 (NC): Animals were administered with olive oil (intraperitoneal injection; 0.25 ml/kg; every 3rd day for 9 weeks) and 0.25% Na-CMC, Group 2 (DC): Animals were administered with CCl_4_ in olive oil v/v (intraperitoneal injection; 0.5 ml/kg; every 3rd day for 9 weeks), Group 3 (PC): Animals were administered with CCl_4_ in olive oil v/v (intraperitoneal injection; 0.5 ml/kg; every 3rd day for 9 weeks) with the concurrent oral treatment of SLM (100 mg/kg; once daily for 9 weeks), Group 4 (SKK-treated): Animals were administered with CCl_4_ in olive oil v/v (intraperitoneal injection; 0.5 ml/kg; every 3rd day for 9 weeks) with the concurrent oral treatment of SKK (100 and 200 mg/kg; once daily for 9 weeks). The animals were observed for physiological, histopathological, and biochemical changes during and after completion of the study period. **(B)** Substantial loss of body weight was detected in the CCl_4_ stimulated Wistar rats. Treatment of the CCl_4_ stimulated animals with human equivalent and higher dose of SKK (100 and 200 mg/kg) resulted in the minor recovery in body weight loss. **(C)** Wistar rats treated with CCl_4_ showed loss of food habits over a period of 28 days. Recovery was detected in the CCl_4_ stimulated animals following treatment with the high dose of SKK (200 mg/kg). SLM (100 mg/kg) was used as a positive control in the study and did not induce any changes in the feed habits. Results are expressed as Mean ± Standard Error of Means. One-way analysis of variance (ANOVA) followed by Dunnett's multiple comparison *t*-test was used to calculate the statistical difference. *p*-value ^#^<0.01; *<0.05; **<0.01.

**Figure 6:**
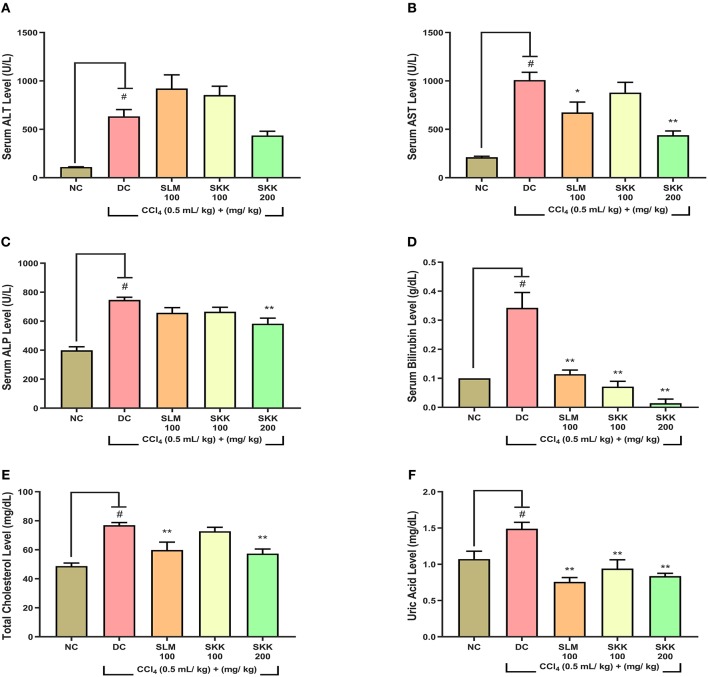
*In-vivo* biochemical analysis of CCl_4_ stimulated wistar rats treated with Divya Sarva-Kalp-Kwath (SKK): Wistar rats stimulated with CCl_4_ showed a significant upsurge in their serum enzyme levels–**(A)** alanine transaminase (ALT), **(B)** aspartate aminotransferase (AST), **(C)** alkaline phosphatase (ALP), **(D)** total bilirubin, **(E)** total cholesterol, and **(F)** uric acid. Co-treatment of the CCl_4_ stimulated rats with SKK (100 and 200 mg/kg) showed a significant reduction in their serum liver injury biomarkers. SLM (100 mg/kg) was used as a positive control in the study. Results are expressed as Mean ± Standard Error Mean. One-way analysis of variance (ANOVA) followed by Dunnett's multiple comparison *t*-test was used to calculate the statistical difference. *p*-value ^#^<0.01; *<0.05; **<0.01.

Liver injury was induced in the male Wistar rats using an intraperitoneal injection of CCl_4_ at the dose of 0.5 mg/kg. A variety of physiological, biochemical, and histopathological markers were tested during the study (Figure 5A). Compared to the NC animals, the disease control (DC) animals showed a significant decline (*p* < 0.01) in their body weights (Figure 5B). Treatment of the CCl_4_ stimulated animals with human equivalent dose (100 mg/kg), and a higher dose (200 mg/kg) of SKK displayed significant (*p* < 0.01) recovery of their body weight (Figure 5B). This SKK induced recovery was comparable to those induced by SLM (100 mg/kg). Following, a 6 weeks' treatment of the CCl_4_ stimulated rats, significant recovery was observed in the feeding habit of the animals following treatment with the 200 mg/kg dose of SKK decoction. This recovery was found to be stable until the end of the 9 weeks' study period. Further, animals treated with SKK at the concentration of 100 mg/kg and reference drug, SLM showed no change in their feed consumption (Figure 5C). Similar to the observations made under *in-vitro* conditions, Wistar rats showed a significant (*p* < 0.01) elevation in their serum levels of ALT, AST, ALP, bilirubin, total cholesterol, and uric acid following stimulation with CCl_4_ (Figure 6). Enhanced levels of these serum biomarkers indicated CCl_4_ induced injury to the liver and kidney of the stimulated animals. Treatment of the CCl_4_ pre-treated rats with equivalent concentration (100 mg/kg) of SKK or, SLM showed no alteration in the release of ALT enzyme (Figure 6A). Treatment of the CCl_4_ stimulated rats with a higher concentration of SKK (200 mg/kg) showed a considerable but statistically non-significant reduction in ALT levels in comparison to the DC animals. The CCl_4_ stimulated increase in the AST levels of rats, was significantly reduced following treatment of the animals with SKK (200 mg/kg; *p* < 0.01) and the reference drug SLM (100 mg/kg; *p* < 0.05) (Figure 6B). Serum ALP levels also showed a significant (*p* < 0.05) reduction in the CCl_4_ stimulated rats following treatment with SKK (200 mg/kg) (Figure 6C). Total bilirubin, cholesterol, and uric acids were also prominently reduced in the CCl_4_ stimulated rats following treatments with the different concentrations of SKK or SLM (Figures 6D–F).

**Figure 7:**
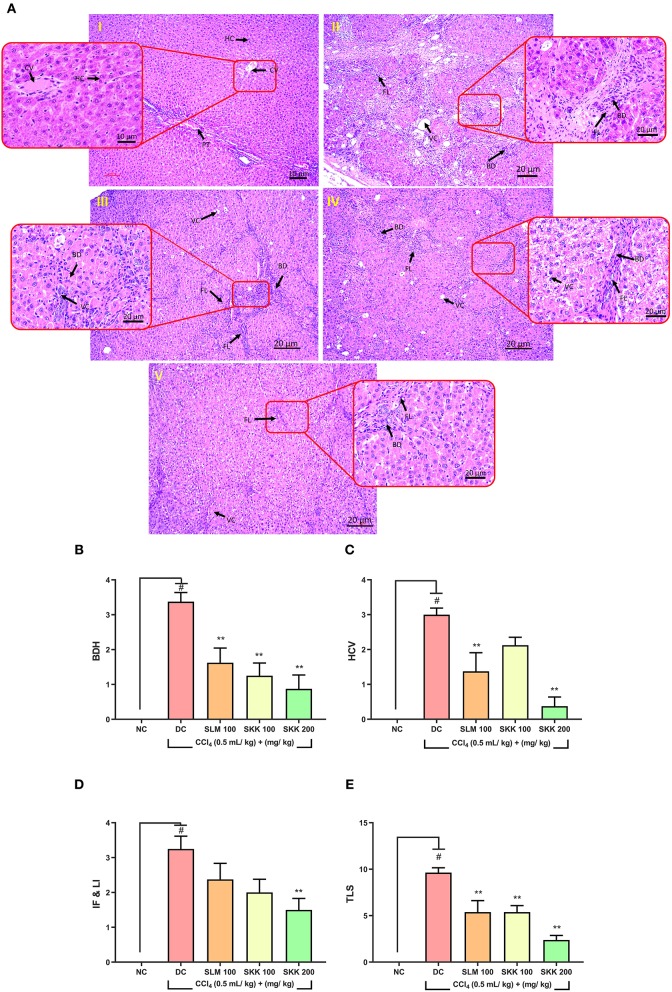
Histopathological Analysis of Wistar Rats Treated with CCl_4_ and Co-Treated with Divya Sarva-Kalp-Kwath (SKK): **(A)** Histopathological investigation was performed in the liver tissue samples obtained from the Wistar rats treated for 9 weeks with CCl_4_, reference drug: SLM (100 mg/kg) and SKK (100 and 200 mg/kg). Results indicated- (i) Untreated animal liver tissue represents normal histology of liver tissue; portal triad (PT), central vein (CV), hepatocyte, (ii) CCl_4_ treated animals showed the presence of severe fibrosis and lymphocytic infiltration (FL), hyperplastic bile duct in the hepatic regions, (iii) Wistar rats treated with SLM (100 mg/kg) showed the development of moderate fibrosis and lymphocytic infiltration (FL), hyperplastic bile duct (BD), vacuolation (VC) in the hepatic tissues; (iv) CCl_4_ and SKK (100 mg/kg) co-treated animals showed moderate levels of fibrosis and lymphocytic infiltration (FL), hyperplastic bile duct (BD), vacuolation (VC) in their hepatic region; (v) Wistar rats treated with CCl_4_ and higher dose of SKK (200 mg/kg) showed the presence of mild fibrosis and lymphocytic infiltration (FL), hyperplastic bile duct (BD) in their hepatic regions. Finally, based on individual lesion scoring, the CCl_4_ treated animals showed marked increase in- **(B)** hyperplastic bile duct (BDH), **(C)** Hepatocellular vacuolation (HCV), **(D)** interlobular fibrosis and lymphocytic infiltration (IF & LI), and **(E)** Total Lesion Score (TLS). The positive control drug, SLM (100 mg/kg) significantly ameliorated the hepatic injuries induced by CCl_4_ treatment. CCl_4_ stimulated animals co-treated with SKK at 100 and 200 mg/kg also showed significant amelioration in their hepatic lesion score. Results are expressed as Mean ± Standard Error of Means. One-way analysis of variance (ANOVA) followed by Dunnett's multiple comparison *t*-test was used to calculate the statistical difference. *p*-value ^#^<0.01; **<0.01.

Histopathological analysis of the liver tissue obtained from the euthanized CCl_4_ stimulated Wistar rats showed the development of inflammatory fibrosis, lymphocytic infiltration, and hyperplastic bile duct (Figure 7Ai,ii). Treatment of the CCl_4_ stimulated rats with SKK (100 and 200 mg/kg) or SLM (100 mg/kg) displayed a remarkable reduction in the severity and distribution of these inflammatory lesions in the liver tissue viz. moderate fibrosis, lymphocytic infiltration, hyperplastic bile duct, and vacuolation (Figure 7Aiii–v). Based on the lesion score criteria, CCl_4_ stimulated rats showed an approximately 10-fold increase in their “total lesion score” (Figure 7E). Further, treatment with SKK at 100 and 200 mg/kg significantly (*p* < 0.01) reduced the “total lesion score” in a dose-dependent manner (Figure 7E). Reference drug, SLM (100 mg/kg) treatment showed a significant (*p* < 0.01) reduction in “total lesion score” as compared to DC animals.

Individual lesion score analysis, further confirmed the histopathological observations, showing significant induction (*p* < 0.01) of bile duct hyperplasia, hepatocellular vacuolation, interlobular fibrosis, and lymphocytic infiltrations, in the DC animals. Treatment of the stimulated animals with SKK (200 mg/kg) showed signs of significant (*p* < 0.01) improvements in CCl_4_ induced injuries through reduction of the inflammatory lesions. Low dose treatment of the stimulated animals with SKK (100 mg/kg) could only lessen the induced bile duct hyperplasia (*p* < 0.01). Similarly, reference drug SLM (100 mg/kg) displayed prominent (*p* < 0.01) reduction in the induced bile duct hyperplasia, hepatocellular vacuolation, interlobular fibrosis (IF), and lymphocytic infiltration (Figures 7B–E).

It is interesting to note that in the overall study, we could observe that the treatment of CCl_4_ stimulated rats with both the concentrations of SKK (100 and 200 mg/kg) and reference drug- SLM (100 mg/kg) significantly reduced the liver injuries. The human equivalent dose of SKK (100 mg/kg) in several parameters showed an equivalent pharmacological effect to SLM (100 mg/kg). However, a higher dose of SKK (200 mg/kg) showed a better hepatoprotective effect than the SLM, against the CCl_4_ induced hepatic damages.

## Discussion

CCl_4_ is well-documented for inducing hepatotoxicity in both animals and humans and extensively used as a study model for evaluating the pharmacological effects of newly synthesized drugs and herbal formulations (Manibusan et al., 2007; Ingawale et al., 2014; Dong et al., 2016). The three herbal components of SKK decoction (*B. diffusa* L., *P. niruri* L., and *S. nigrum* L.) are well-known to have anti-oxidant, anti-inflammatory, and hepatoprotective properties (Lin et al., 2008; Manjrekar et al., 2008; Ramachandra et al., 2011).

Plant metabolites play a major role in the active prevention of diseases. Hence, our chemical analysis of the SKK decoction revealed the presence of several plant metabolites such as rotenoids (boeravinones: A, B, E, G, I, K), eupalitin 3-galactoside, eupalitin, coccineone B, koaburaside, pyrogallic acid, 1-O-caffeoyquinic acid, phyllanthusiin E, corilagin, gallocatechin, astragalin, tartaric acid, gentisic acid, 3-O-trans-coumaroylquinic acid, protocatechuic aldehyde, 5-caffeoylquinic acid, caffeic acid, m-coumaric acid, citrusin B, myricitrin, solamargine, naringenin, rhoifolin, apigenin, solasonin, solanine, solasodine 3-b-D-glucopyranoside, butein, solasodine, and tigogenin. Based on literature search, the origin of these plant metabolites was identified from *B. diffusa* L., *P.niruri* L., and *S. nigrum* L. (Pandey et al., 2005; Bairwa et al., 2013; Mishra et al., 2014; Sarin et al., 2014; Jantan et al., 2019). Other plant metabolites identified through the chemical analysis were common to the three plant species.

In our 28 days' chronic safety study, SKK did not induce any form of toxicity in the Wistar rats following repeated dosing up to the highest tested concentration of 1,000 mg/kg (10× human equivalent dose for rats). Earlier sub-acute safety study of SKK had reported similar findings where the authors calculated the lethal-dose 50% of SKK at concentration >2,000 mg/kg (Yadav and Kumar, 2014). Toxicity studies performed on the individual plant extract of *B. diffusa* L. and *P. niruri* L. have reported them to be non-toxic in rats (Asare et al., 2011). Raw plant materials obtained from *S. nigrum* L. have been reported to have high toxicity in humans and cattle. However, pre-treatment of these plant parts on boiling (decocting) can render them non-toxic (Kuete, 2013; Mishra et al., 2014).

*In-vitro* and *in-vivo* treatment of the CCl_4_ stimulated human hepatocyte (HepG2 cells) and Wistar rats, with SKK decoction, showed noteworthy hepatoprotective effects through the amelioration of cytotoxicity, generation of ROS, down-regulation of MMP and reduction in the release of AST, ALP, bilirubin, and total cholesterol. Compared to the positive control drug SLM (100 mg/kg), low (100 mg/kg), and high (200 mg/kg) dose treatments of SKK showed sizable pharmacological effects in ameliorating CCl_4_ stimulated hepatic injuries in the Wistar rats. Uric acid level recovery in the CCl_4_ stimulated rats also indicated a vital role for SKK in providing kidney protection against chemical-induced injury. Our study showed a good correlation with the earlier short-term (7 days), sub-acute, single-dose (120 mg/kg) exposure study performed by Yadav and Kumar (2014). They also observed that the SKK decoction treatment of Wistar rats inhibited CCl_4_ induced hepatotoxicity and inhibited the release of serum ALT, ALP, bilirubin, and albumin levels (Yadav and Kumar, 2014). However, it is interesting to observe that the low dose of SKK (100 mg/kg) applied in our chronic, long-term study did not perform well compared to the sub-acute study performed by Yadav and Kumar (2014). Hence, from our study, it can be inferred that under chronic exposure to CCl_4_ inducing severe hepatic injuries, SKK is required at a higher dose of ~200 mg/kg in Wistar rats.

Finally, the hepatoprotective effect of SKK can be attributed to the presence of plant metabolites detected using the LC-MS QToF analytical method. Several of the detected plant metabolites have been reported to have excellent antioxidant, anti-inflammatory and hepatoprotective properties (Pandey et al., 2005; Bairwa et al., 2013; Mishra et al., 2014; Sarin et al., 2014; Jantan et al., 2019). Rotenoids (boeravinones) also possess anti-oxidant properties as they have been observed to have inhibitory effects on the production of cyclooxygenase (COX)-1, COX-2, and generation of cellular reactive oxygen species (Aviello et al., 2011; Bairwa et al., 2013; Son and Phan, 2014). Six plant metabolites further quantified using HPLC (caffeic acid, rutin, gallic acid, catechin, quercetin, and corilagin) have been studied extensively for their hepatoprotective activities against CCl_4_ and drug exposures (Janbaz et al., 2004; Wang et al., 2014, 2019; Elsawy et al., 2019; Ezzat et al., 2020). Owing to the different mode of action for the three plants *per se* and the presence of large variety of plant metabolites, it can be speculated that SKK probably functions at multiple levels in inhibiting CCl_4_ induced hepatic injuries, i.e., through inhibition of oxidative stress, pro-inflammatory responses, and metabolizing enzymes CYPs (Khan et al., 2012; Wang et al., 2014; Ekstrand et al., 2015; Li et al., 2018). Finally, the presence of three herbal components also produced a possible synergistic effect in ameliorating CCl_4_ induced hepatic injury that needs to be explored further. The dose of individual plant extract applied at the highest tested dose of SKK (200 mg/kg) was relatively low as compared to several other published studies using individual plant extracts of *B. diffusa* L. *, P. niruri* L., and *S. nigrum* L. for hepatoprotection in CCl_4_ stimulated murine models (Chandan et al., 1991; Manjrekar et al., 2008; Patel et al., 2014; Beedimani and Jeevangi, 2017; Krithika and Verma, 2019).

## Conclusion

Finally, the present work revealed that SKK has prominent pharmacological effects against long-term, chronic CCl_4_ induced liver damages in Wistar rats and in human hepatocytes. Therefore, it is important to perform further experiments to understand its mode of action. This would reconfirm the overall biological effect of “Divya Sarva-Kalp-Kwath” medicine as the potent hepatoprotective therapeutics.

## Data Availability Statement

All datasets generated for this study are included in the article/supplementary material.

## Ethics Statement

The animal study was reviewed and approved by Institutional Animal Ethical Committee of the Patanjali Research Institute, Haridwar, India.

## Author Contributions

AB provided broad direction for the study, identified the test formulation, generated resources, and gave final approval for the manuscript. SSS did the *in-vivo* study execution, and data analysis. RR performed the *in-vitro* experiments and manuscript reviewing. KhJ assisted in performing *in-vivo* studies. SS executed the safety studies. KaJ prepared the histopathological slides. SV performed the LC-MS analysis. AG analyzed the data. KB performed *in-vitro* experiments, wrote, and revised the overall manuscript. SSS, SS, RR, SV, AG, KB, and AV contributed in final manuscript revision. AV supervised overall research project planning, generated resources, reviewed, and finally approved the manuscript.

### Conflict of Interest

The authors declare that the research was conducted in the absence of any commercial or financial relationships that could be construed as a potential conflict of interest.

## References

[B1] Abraham P. Wilfred G. (2002). A massive increase in serum beta-glucuronidase after a single dose of carbon tetrachloride to the rat. Clin. Chim. Acta 322, 183–184. 10.1016/s0009-8981(02)00170-5 12104100

[B2] Albouchi F. Attia M. Hanana M. Hamrouni L. (2018). Ethnobotanical notes and phytopharmacologiques on *Solanum nigrum* Linn. (Family: Solanaceae). Am. J. Phytomed. Clin. Ther. 6:5 10.21767/2321-2748.100341

[B3] Asare G. A. Addo P. Bugyei K. Gyan B. Adjei S. Otu-Nyarko L. S.. (2011). Acute toxicity studies of aqueous leaf extract of *Phyllanthus niruri*. Interdiscip. Toxicol. 4, 206–210. 10.2478/v10102-011-0031-9 22319255 PMC3274729

[B4] Atanu F. Ebiloma G. Ajayi E. (2011). A review of the pharmacological aspects of *Solanum nigrum* Linn. Biotechnol. Mol. Biol. Rev. 6, 1–7.

[B5] Aviello G. Canadanovic-Brunet J. M. Milic N. Capasso R. Fattorusso E. Taglialatela-Scafati O.. (2011). Potent antioxidant and genoprotective effects of boeravinone G, a rotenoid isolated from Boerhaavia diffusa. PLoS ONE 6:e19628. 10.1371/journal.pone.0019628 21625488 PMC3098844

[B6] Bairwa K. Singh I. N. Roy S. K. Grover J. Srivastava A. Jachak S. M. (2013). Rotenoids from Boerhaavia diffusa as potential anti-inflammatory agents. J. Nat. Prod. 76, 1393–1398. 10.1021/np300899w 23914900

[B7] Balkrishna A. Pokhrel S. Tomer M. Verma S. Kumar A. Nain P.. (2019a). Anti-Acetylcholinesterase activities of mono-herbal extracts and exhibited synergistic effects of the phytoconstituents: a biochemical and computational study. Molecules 24:4175. 10.3390/molecules24224175 31752124 PMC6891289

[B8] Balkrishna A. Sakat S. S. Joshi K. Joshi K. Sharma V. Ranjan R.. (2019b). Cytokines driven anti-inflammatory and anti-psoriasis like efficacies of nutraceutical sea buckthorn (*Hippophae rhamnoides*) Oil. Front. Pharmacol. 10:1186. 10.3389/fphar.2019.01186 31680964 PMC6797847

[B9] Balkrishna A. Sakat S. S. Joshi K. Paudel S. Joshi D. Joshi K. (2019c). Anti-inflammatory and anti-arthritic efficacies of an indian traditional herbo-mineral medicine “divya amvatari ras” in collagen antibody-induced arthritis (CAIA) mouse model through modulation of IL-6/IL-1beta/TNF-alpha/NFkappaB signaling. Front. Pharmacol. 10:659 10.3389/fphar.2019.00659 31333447 PMC6614787

[B10] Balkrishna A. Sakat S. S. Joshi K. Paudel S. Joshi D. Joshi K.. (2019d). Herbo-mineral formulation 'Ashwashila' attenuates rheumatoid arthritis symptoms in collagen-antibody-induced arthritis (CAIA) mice model. Sci. Rep. 9:8025. 10.1038/s41598-019-44485-9 31142786 PMC6541602

[B11] Beedimani R. S. Jeevangi S. K. (2017). Evaluation of hepatoprotective activity of Boerhaavia diffusa against carbon tetrachloride induced liver toxicity in albino rats. Int. J. Basic Clin. Pharmacol. 4:153 10.5455/2319-2003.ijbcp20150230

[B12] Bhattacharjee R. Sil P. C. (2007). Protein isolate from the herb *Phyllanthus niruri* modulates carbon tetrachloride-induced cytotoxicity in hepatocytes. Toxicol. Mech. Methods 17, 41–47. 10.1080/15376510600970034 20020986

[B13] Borrelli F. Milic N. Ascione V. Capasso R. Izzo A. A. Capasso F.. (2005). Isolation of new rotenoids from Boerhaavia diffusa and evaluation of their effect on intestinal motility. Planta Med. 71, 928–932. 10.1055/s-2005-871282 16254824

[B14] Bovy A. Schijlen E. Hall R. D. (2007). Metabolic engineering of flavonoids in tomato (*Solanum lycopersicum*): the potential for metabolomics. Metabolomics 3, 399–412. 10.1007/s11306-007-0074-2 25653576 PMC4309898

[B15] Chandan B. K. Sharma A. K. Anand K. K. (1991). Boerhaavia diffusa: a study of its hepatoprotective activity. J. Ethnopharmacol. 31, 299–307. 10.1016/0378-8741(91)90015-6 2056758

[B16] Daji G. Steenkamp P. Madala N. Dlamini B. (2018). Phytochemical composition of *Solanum retroflexum* analysed with the aid of ultra-performance liquid chromatography hyphenated to quadrupole-time-of-flight mass spectrometry (UPLC-qTOF-MS). J. Food Qual. 2018:8 10.1155/2018/3678795

[B17] De Souza G. R. De-Oliveira A. C. A. X. Soares V. Chagas L. F. Barbi N. S.. (2019). Chemical profile, liver protective effects and analgesic properties of a *Solanum paniculatum* leaf extract. Biomed. Pharmacother. 110, 129–138. 10.1016/j.biopha.2018.11.036 30466002

[B18] Debnath S. Ghosh S. Hazra B. (2013). Inhibitory effect of *Nymphaea pubescens* Willd. flower extract on carrageenan-induced inflammation and CCl(4)-induced hepatotoxicity in rats. Food Chem. Toxicol. 59, 485–491. 10.1016/j.fct.2013.06.036 23827777

[B19] Dong S. Chen Q. L. Song Y. N. Sun Y. Wei B. Li X. Y.. (2016). Mechanisms of CCl4-induced liver fibrosis with combined transcriptomic and proteomic analysis. J. Toxicol. Sci. 41, 561–572. 10.2131/jts.41.561 27452039

[B20] Ekow Thomford N. Dzobo K. Adu F. Chirikure S. Wonkam A. Dandara C. (2018). Bush mint (*Hyptis suaveolens*) and spreading hogweed (*Boerhavia diffusa*) medicinal plant extracts differentially affect activities of CYP1A2, CYP2D6 and CYP3A4 enzymes. J. Ethnopharmacol. 211, 58–69. 10.1016/j.jep.2017.09.023 28942133

[B21] Ekstrand B. Rasmussen M. K. Woll F. Zlabek V. Zamaratskaia G. (2015). *In vitro* gender-dependent inhibition of porcine cytochrome p450 activity by selected flavonoids and phenolic acids. Biomed. Res. Int. 2015:387918. 10.1155/2015/387918 25685784 PMC4317639

[B22] Elhag R. A. M. Sma E. B. Bakhiet A. O. Galal M. (2011). Hepatoprotective activity of Solanum nigrum extracts on chemically induced liver damage in rats. J. Vet. Med. Ani. Health 3, 45–50.

[B23] Elsawy H. Badr G. M. Sedky A. Abdallah B. M. Alzahrani A. M. Abdel-Moneim A.M. (2019). Rutin ameliorates carbon tetrachloride (CCl4)-induced hepatorenal toxicity and hypogonadism in male rats. Peer J. 7:e7011. 10.7717/peerj.7011 31179192 PMC6545103

[B24] Ezzat M. I. Okba M. M. Ahmed S. H. El-Banna H. A. Prince A. Mohamed S. O.. (2020). In-depth hepatoprotective mechanistic study of *Phyllanthus niruri: in vitro* and *in vivo* studies and its chemical characterization. PLoS ONE 15:e0226185. 10.1371/journal.pone.0226185 31940365 PMC6961881

[B25] Ferreres F. Sousa C. Justin M. Valentão P. Andrade P. B. Llorach R.. (2005). Characterisation of the phenolic profile of Boerhaavia diffusa L. by HPLC-PAD-MS/MS as a tool for quality control. Phytochem. Anal. 16, 451–458. 10.1002/pca.869 16315490

[B26] Harish R. Shivanandappa T. (2006). Antioxidant activity and hepatoprotective potential of *Phyllanthus niruri*. Food Chem. 95, 180–185. 10.1016/j.foodchem.2004.11.049

[B27] Huang H. C. Syu K. Y. Lin J. K. (2010). Chemical composition of *Solanum nigrum* linn extract and induction of autophagy by leaf water extract and its major flavonoids in AU565 breast cancer cells. J. Agric Food Chem. 58, 8699–8708. 10.1021/jf101003v 20681660

[B28] Ingawale D. K. Mandlik S. K. Naik S. R. (2014). Models of hepatotoxicity and the underlying cellular, biochemical and immunological mechanism(s): a critical discussion. Environ. Toxicol. Pharmacol. 37, 118–133. 10.1016/j.etap.2013.08.015 24322620

[B29] Janbaz K. H. Saeed S. A. Gilani A. H. (2004). Studies on the protective effects of caffeic acid and quercetin on chemical-induced hepatotoxicity in rodents. Phytomedicine 11, 424–430. 10.1016/j.phymed.2003.05.002 15330498

[B30] Jantan I. Haque M. A. Ilangkovan M. Arshad L. (2019). An insight into the modulatory effects and mechanisms of action of phyllanthus species and their bioactive metabolites on the immune system. Front. Pharmacol. 10:878. 10.3389/fphar.2019.00878 31440162 PMC6693410

[B31] Juneja K. Mishra R. Chauhan S. Gupta S. Roy P. Sircar D.. (2020). Metabolite profiling and wound-healing activity of Boerhavia diffusa leaf extracts using *in vitro* and *in vivo* models. J. Trad. Comp. Med. 10, 52–59. 10.1016/j.jtcme.2019.02.002 31956558 PMC6957803

[B32] Kaunda J. S. Zhang Y.-J. (2019). The genus solanum: an ethnopharmacological, phytochemical and biological properties review. Nat. Prod. Bioprosp. 9:6. 10.1007/s13659-019-0201-6 30868423 PMC6426945

[B33] Kaur N. Kaur B. Sirhindi G. (2017). Phytochemistry and pharmacology of *Phyllanthus niruri* L.: a review. Phytother. Res. 31, 980–1004. 10.1002/ptr.5825 28512988

[B34] Khan R. A. Khan M. R. Sahreenm S. (2012). CCl4-induced hepatotoxicity: protective effect of rutin on p53, CYP2E1 and the antioxidative status in rat. BMC Complement Altern. Med. 12:178. 10.1186/1472-6882-12-178 23043521 PMC3519517

[B35] Kirtikar K. R. Basu B. D. (1956). Indian Medicinal Plants. Allahabad: Lalit Mohan Basu.

[B36] Krithika R. Verma R. J. (2019). *Solanum nigrum* confers protection against CCl4-induced experimental hepatotoxicity by increasing hepatic protein synthesis and regulation of energy metabolism. Clin. Phytosci. 5:1 10.1186/s40816-018-0096-5

[B37] Kuete V. (2013). Physical, Hematological, and Histopathological Signs of Toxicity Induced by African Medicinal Plants. Oxford: Elsevier 10.1016/B978-0-12-800018-2.00022-4

[B38] Kumar S. Singh A. Singh B. Maurya R. Kumar B. (2018). Structural characterization and quantitative determination of bioactive compounds in ethanolic extracts of *Boerhaavia diffusa* L. by liquid chromatography with tandem mass spectrometry. Sep. Sci. Plus 1, 588–596. 10.1002/sscp.201800056

[B39] Lami N. Kadota S. Tezuka Y. Kikuchi T. (1990). Constituents of the roots of Boerhaavia diffusa Linn. IV. Isolation and structure determination of boeravinones D, E and F. Chem. Pharm. Bull. 38, 1558–1562. 10.1248/cpb.38.1558

[B40] Li X. Deng Y. Zheng Z. Huang W. Chen L. Tong Q.. (2018). Corilagin, a promising medicinal herbal agent. Biomed Pharmacother. 99, 43–50. 10.1016/j.biopha.2018.01.030 29324311

[B41] Li Y. Chang W. Zhang M. Ying Z. Lou H. (2015). Natural product solasodine-3-O-beta-D-glucopyranoside inhibits the virulence factors of Candida albicans. FEMS Yeast Res. 15:fov060. 10.1093/femsyr/fov060 26162798

[B42] Lin H. M. Tseng H. C. Wang C. J. Lin J. J. Lo C. W. Chou F. P. (2008). Hepatoprotective effects of *Solanum nigrum* Linn extract against CCl(4)-induced oxidative damage in rats. Chem. Biol. Interact. 171, 283–293. 10.1016/j.cbi.2007.08.008 18045581

[B43] Manibusan M. K. Odin M. Eastmond D. A. (2007). Postulated carbon tetrachloride mode of action: a review. J. Environ. Sci. Health C Environ. Carcinog. Ecotoxicol. Rev. 25, 185–209. 10.1080/10590500701569398 17763046

[B44] Manjrekar A. P. Jisha V. Bag P. P. Adhikary B. Pai M. M. Hegde A.. (2008). Effect of *Phyllanthus niruri* Linn. treatment on liver, kidney and testes in CCl4 induced hepatotoxic rats. Indian J. Exp. Biol. 46, 514–520. 18807755

[B45] Mao X. Wu L. F. Guo H. L. Chen W. J. Cui Y. P. Qi Q.. (2016). The genus phyllanthus: an ethnopharmacological, phytochemical, and pharmacological review. Evid. Based Compl. Alternat. Med. 2016:7584952. 10.1155/2016/7584952 27200104 PMC4854999

[B46] Mir A. Anjum F. Riaz N. Iqbal H. Wahedi H. M. Khattak J. Z. K. (2010). Carbon tetrachloride (CCl4) - induced hepatotoxicity in rats: curative role of *Solanum nigrum*. J. Med. Plants Res. 4, 2525–2532. 10.5897/JMPR10.482

[B47] Mishra S. Aeri V. Gaur P. K. Jachak S. M. (2014). Phytochemical, therapeutic, and ethnopharmacological overview for a traditionally important herb: *Boerhavia diffusa* Linn. Biomed. Res. Int. 2014:808302. 10.1155/2014/808302 24949473 PMC4053255

[B48] Nada S. A. Omara E. A. Abdel-Salam O. M. Zahran H. G. (2010). Mushroom insoluble polysaccharides prevent carbon tetrachloride-induced hepatotoxicity in rat. Food Chem. Toxicol. 48, 3184–3188. 10.1016/j.fct.2010.08.019 20732378

[B49] Nair A. B. Jacob S. (2016). A simple practice guide for dose conversion between animals and human. J. Basic Clin. Pharm. 7:27. 10.4103/0976-0105.177703 27057123 PMC4804402

[B50] Pandey R. Maurya R. Singh G. Sathiamoorthy B. Naik S. (2005). Immunosuppressive properties of flavonoids isolated from *Boerhaavia diffusa* Linn. Int. Immunopharmacol. 5, 541–553. 10.1016/j.intimp.2004.11.001 15683850

[B51] Patel A. Biswas S. Shoja M. H. Ramalingayya G. V. Nandakumar K. (2014). Protective effects of aqueous extract of *Solanum nigrum* Linn. leaves in rat models of oral mucositis. Sci. World J. 2014:10. 10.1155/2014/345939 25506066 PMC4258331

[B52] Patel M. Verma R. (2014). Hepatoprotective activity of *Boerhavia diffusa* extract. Int. J. Pharm. Clin. Res. 6, 233–240.

[B53] Pereira D. M. Faria J. Gaspar L. Valentão P. De Pinho P. G. Andrade P. B. (2009). *Boerhaavia diffusa*: metabolite profiling of a medicinal plant from Nyctaginaceae. Food Chem. Toxicol. 47, 2142–2149. 10.1016/j.fct.2009.05.033 19500634

[B54] Ramachandra Y. L. Shilali K. Ahmed M. Sudeep H. V. Kavitha B. T. Gurumurthy H. (2011). Hepatoprotective properties of *Boerhavia diffusa* and aerva lanata against carbon tetra chloride induced hepatic damage rats. Pharmacology Online 3, 435–441.

[B55] Refaat J. Yehia S. Ramadan M. Kamel M. (2015). Rhoifolin: a review of sources and biological activities. Int. J. Pharmacog. 2, 102–109.

[B56] Sarin B. Verma N. Martín J. P. Mohanty A. (2014). An overview of important ethnomedicinal herbs of Phyllanthus species: present status and future prospects. Sci. World J. 2014:839172. 10.1155/2014/839172 24672382 PMC3932249

[B57] Sivgami S. Gayathri P. Ramapriya R. (2012). The antioxidant potential of two selected varieties of *Solanum nigrum*. J. Pharm. Res. 5, 2221–2223.

[B58] Son H. Phan Y. (2014). Preliminary phytochemical screening, acute oral toxicity and anticonvulsant activity of the berries of *Solanum nigrum* Linn. Trop. J. Pharm. Res. 13:907 10.4314/tjpr.v13i6.12

[B59] Syamasundar K. V. Singh B. Thakur R. S. Husain A. Kiso Y. Hikino H. (1985). Antihepatotoxic principles of *Phyllanthus niruri* herbs. J. Ethnopharmacol. 14, 41–44. 10.1016/0378-8741(85)90026-1 4087921

[B60] Venkatesh P. Dinakar A. Senthilkumar N. (2012). Hepatoprotective activity of alcoholic extracts of *Boerhaavia diffusa* and Anisochlilus Carnosus against carbon tetrachloride induced hepatotoxicity in rats. Asian J. Pharm. Clin. Res. 5, 232–234.

[B61] Wang J. Tang L. White J. Fang J. (2014). Inhibitory effect of gallic acid on CCl4-mediated liver fibrosis in mice. Cell Biochem. Biophys. 69, 21–26. 10.1007/s12013-013-9761-y 24096707

[B62] Wang L. Yang G. Yuan L. Yang Y. Zhao H. Ho C. T.. (2019). Green tea catechins effectively altered hepatic fibrogenesis in rats by inhibiting ERK and Smad1/2 phosphorylation. J. Agric Food Chem. 67, 5437–5445. 10.1021/acs.jafc.8b05179 30424599

[B63] Williamson E. M. (2001). Synergy and other interactions in phytomedicines. Phytomedicine 8, 401–409. 10.1078/0944-7113-00060 11695885

[B64] Yadav A. Kumar S. (2014). Hepatoprotective effect of sarvakalp kwath against carbon tetrachloride induced hepatic injury in albino rats. Biomed. Pharmacol. J. 7, 659–663. 10.13005/bpj/538

[B65] Yadav N. P. Pal A. Shanker K. Bawankule D. U. Gupta A. K. Darokar M. P.. (2008). Synergistic effect of silymarin and standardized extract of Phyllanthus amarus against CCl4-induced hepatotoxicity in *Rattus norvegicus*. Phytomedicine 15, 1053–1061. 10.1016/j.phymed.2008.08.002 18848770

[B66] Zhou X. Seto S. W. Chang D. Kiat H. Razmovski-Naumovski V. Chan K.. (2016). Synergistic effects of chinese herbal medicine: a comprehensive review of methodology and current research. Front. Pharmacol. 7:201. 10.3389/fphar.2016.00201 27462269 PMC4940614

